# LORETA With Cortical Constraint: Choosing an Adequate Surface Laplacian Operator

**DOI:** 10.3389/fnins.2018.00746

**Published:** 2018-10-30

**Authors:** Todor Iordanov, Harald Bornfleth, Carsten H. Wolters, Vesela Pasheva, Georgi Venkov, Benjamin Lanfer, Michael Scherg, Tobias Scherg

**Affiliations:** ^1^BESA GmbH, Gräfelfing, Germany; ^2^Institute for Biomagnetism and Biosignalanalysis, University of Münster, Münster, Germany; ^3^Faculty of Applied Mathematics and Informatics, Technical University of Sofia, Sofia, Bulgaria

**Keywords:** Algorithms, Brain imaging, Electroencephalography, Magnetoencephalopgraphy, cortical imaging, surface Laplacian, Brain Mapping

## Abstract

Low resolution electromagnetic tomography (LORETA) is a well-known method for the solution of the l2-based minimization problem for EEG/MEG source reconstruction. LORETA with a volume-based source space is widely used and much effort has been invested in the theory and the application of the method in an experimental context. However, it is especially interesting to use anatomical prior knowledge and constrain the LORETA's solution to the cortical surface. This strongly reduces the number of unknowns in the inverse approach. Unlike the Laplace operator in the volume case with a rectangular and regular grid, the mesh is triangulated and highly irregular in the surface case. Thus, it is not trivial to choose or construct a Laplace operator (termed Laplace-Beltrami operator when applied to surfaces) that has the desired properties and takes into account the geometry of the mesh. In this paper, the basic methodology behind cortical LORETA is discussed and the method is applied for source reconstruction of simulated data using different Laplace-Beltrami operators in the smoothing term. The results achieved with the different operators are compared with respect to their accuracy using various measures. Conclusions about the choice of an appropriate operator are deduced from the results.

## Introduction

Neuroscience is a fast growing field posing many challenges and requiring expertise in various different scientific areas (Markram, 2013). In order to be able to satisfy these needs the neuroscience community is asked to constantly develop new strategies for data analysis, design new experiments, improve techniques used in the past, identify previous errors and correct them etc. Where neurophysiological measurements are concerned, at the beginning of the electroencephalographic (EEG) era (Berger, 1929) scientists and physicians were content with looking merely at the signals measured on the scalp. However, a few decades later the question on where in the brain these signals originate gained more relevance (Brazier, 1949; Wilson and Bayley, 1950). This was the time when source reconstruction of brain activity was born. From then onwards, strategies and methods of source analysis were rapidly developing and many different approaches came into existence. The dipole model was improved to use spatio-temporal information (Scherg and von Cramon, 1985), distributed source models with a linear inverse appeared (Hämäläinen and Ilmoniemi, 1984), their iterative application was discussed (Gorodnitsky et al., 1995), and later also Bayesian methods for source reconstruction (Schmidt et al., 1999) were introduced.

The class of discrete, 3D-distributed, linear inverses contains a large variety of available methods. One very popular and widely used method is low resolution electromagnetic tomography (LORETA) (Pascual-Marqui et al., 1994; Pascual-Marqui, 1999), or also called “Laplacian weighted Minimum Norm” (Michel et al., 2004), which uses the Laplace operator for modeling the source-space correlation of the data. For a critical view on the capabilities of LORETA the reader can refer to (de Peralta Menendez and Andino, 2000). Initially, this method was applied with three-dimensional volume source space without taking into account the structure of the cerebral cortex. Soon after this, an improvement of this method was suggested by constraining the solution to the cortex and using a Laplace operator that smoothes along the two-dimensional cortical surface instead of the 3D brains space (Skrandies et al., 1995). This improvement was suggested earlier for the Minimum norm approach (Dale and Sereno, 1993) and is considered meaningful, since it is based on anatomy and helps to reduce the undesired spreading of the estimated source activity to adjacent gyri or fissures. In the first actual implementation of cortical LORETA it was suggested to use a weighted graph Laplace operator (Wagner et al., 1996). No reason was given for this particular choice of a Laplace operator and the properties of the Laplace operators were not investigated in detail.

In this publication, we introduce four different surface-based Laplace operators which correspond to four different classes of operators. Laplace operators for surfaces are also termed Laplace-Beltrami operators and overviews can be found in Reuter et al. (2009), Dakov and Venkov (2014).

Here, we present results on the unweighted graph Laplacian (Levy, 2006), the weighted graph Laplacian (Wagner et al., 1996), the unweighted geometric Laplacian (Pinkall and Polthier, 1993) and the weighted geometric Laplacian (Meyer et al., 2003) as a special case for a finite elements discretization of the operator (Dziuk, 1988). In the following these four operators are compared with respect to their basic mathematical properties, application and performance in the source reconstruction with cortical LORETA.

## Methods

### Formulation of the LORETA solution

Let *Dϵℝ*^*n* × 1^ be a matrix containing measured EEG data at ***n*** channels for one time sample, and *Lϵℝ*^*n* × 3*m*^ be the lead field matrix which is the solution of the so-called electromagnetic forward problem and contains the information about the data measured at the ***n*** channels if there is a unit strength current dipole at a given position (x, y, z) in the source space. The number of discrete source positions in the brain is denoted by ***m***. *L* can be written in matrix form as:

$$L=\left(\begin{array}{cc} \begin{array}{cc} l_{11}^{T} & l_{12}^{T}\\ l_{21}^{T} & l_{22}^{T} \end{array} & \begin{array}{cc} \cdots & l_{1m}^{T}\\ \cdots & l_{2m}^{T} \end{array}\\ \begin{array}{cc} \vdots & \vdots \\ l_{n1}^{T} & l_{n2}^{T} \end{array} & \begin{array}{cc} & \vdots \\ \cdots & l_{nm}^{T} \end{array} \end{array}\right),$$

where, $l_{ij}={\left(\lambda_{xij},\lambda_{yij},\lambda_{zij}\right)}^{T}\epsilon {\mathbb{R}}^{3\times 1}$ is the lead field for the source location with coordinates (x, y, z).

The problem of EEG source reconstruction using the l2-norm can be formulated as follows:

Find the source current density distribution *J ϵ ℝ*^3*m* × 1^ which satisfies the following condition:

(1) $$\begin{array}{r} \min\left\{||L\cdot J-D|\overset{2}{\underset{2}{|}}+\alpha J^{T}\cdot W\cdot J\right\}. \end{array}$$

Here *W* ϵ ℝ^3*m* × 3*m*^ is the weighting matrix in the regularization term and can be chosen according to the assumed properties of the true solution. If *W* is the identity matrix then we have the original minimum norm solution derived by Hämäläinen and Ilmoniemi (1984), if *W* is a diagonal matrix containing depth weights then the depth-weighted minimum norm is provided. In the case of LORETA the matrix *W* is defined as a combination of the diagonal depth weighting and the discrete Laplace operator. The definition of *W* due to Pascual-Marqui (1999) is given by:

(2) $$\begin{array}{r} W=\left(\Omega B^{T}B\Omega \right)⊗I_{3} \end{array}$$

where $I_{3}\in {\mathbb{R}}^{3\times 3}$ is the 3 × 3 identity matrix, ⊗ is the Kronecker product (Laub, 2005), Ω ϵ ℝ^*m* × *m*^ is a diagonal weighting matrix (Pascual-Marqui, 1999) defined as

(3) $$\begin{array}{r} \Omega_{jj}=\sqrt{\overset{n}{\underset{i=1}{\sum }}l_{ij}^{T}l_{ij}},\;for\;j=1\cdots m \end{array}$$

and *B* ∈ ℝ^*m* × *m*^ is the stiffness matrix of the discrete Laplace operator (Skrandies et al., 1995) defined as

(4) $$\begin{array}{r} \Delta g\left(p_{i}\right)=\frac{6}{d^{2}}\left[\frac{\left(6+\sum_{j\in N\left(i\right)}1\right)}{12\sum_{j\in N\left(i\right)}1}\underset{j\in N\left(i\right)}{\sum }g\left(p_{j}\right)-g\left(p_{i}\right)\right]. \end{array}$$

Here *d* is the distance between two neighboring grid points, *g*(*p*_*i*_) is the value of a function *g* defined on a regular 3D grid at the point *p*_*i*_ from that grid. *N(i)* is the set of indices corresponding to the direct neighbors of *p*_*i*_. B is described below in more detail.

The regularization parameter α > 0 represents the balance between minimizing the residual norm and minimizing the regularization term.

Given the above definitions the solution of the problem (1) is given by:

(5) $$\begin{array}{r} Ĵ=T\cdot D, \end{array}$$

where *T* ∈ ℝ^3*m* × *n*^ is called the linear inverse operator and can be calculated as:

(6) $$\begin{array}{r} T=W^{-1}L^{T}{\left[LW^{-1}L^{T}+\alpha I_{n}\right]}^{-1} \end{array}$$

for a square invertible matrix *W* (Hansen, 1998). The parameter α is the same as in equation (1). The equation (6) is similar to the LORETA solution for volume source space given in Pascual-Marqui (1999). The exact equation for LORETA is

(7) $$\begin{array}{r} T=W^{-1}L^{T}\cdot pinv\left(LW^{-1}L^{T},\beta \mu \right), \end{array}$$

where *pinv* is the pseudoinverse (Björck, 1996) of the matrix *LW*^−1^*L*^*T*^ with tolerance βμ, i.e. setting all singular values of the matrix *LW*^−1^*L*^*T*^ less than the tolerance to zero. This resembles the truncated singular value decomposition introduced to source analysis in Wolters et al. (1999).

Here β is an alternative parameter to α with values also in the interval (0 1]. The parameter μ is the largest singular value of the matrix *LW*^−1^*L*^*T*^.

### LORETA with cortical constraint

The next step is to develop a solution for LORETA on the cerebral cortex, i.e. not for a volume source space but for a two-dimensional surface in the 3D space. First of all, the Laplace operator used before has to be changed to be able to operate on the surface. Suggestions for such Laplace operators were already described in the literature (Skrandies et al., 1995; Wagner et al., 1996). However, in these publications it is not taken into account that a large number of discretizations of the Laplace operator exists on two-dimensional surfaces, with different properties (Wardetzky et al., 2007; Belkin et al., 2008) and, consequently, yielding different results as smoothing operators (Desbrun et al., 1999).

In this publication, four classes of Laplace-Beltrami operators are considered: unweighted graph Laplacians, weighted graph Laplacians, geometric Laplacians without area weights, and geometric Laplacians with area weights.

Let *S* be the continuous cortical surface (2D-manifold) in ℝ^3^. Its discrete form is then

(8) $$\begin{array}{r} M=\left\{p_{i}\epsilon {\mathbb{R}}^{3}|\;p_{i}\epsilon S,\forall i=1,\;\cdots \;,m\right\} \end{array}$$

The general form of the discrete Laplace-Beltrami operator applied to a function *f* on *M* can be written as:

(9) $$\begin{array}{r} \Delta f\left(p_{i}\right)=\frac{1}{d_{i}}\underset{j\in N\left(i\right)}{\sum }w_{ij}\left[f\left(p_{i}\right)-f\left(p_{j}\right)\right] \end{array}$$

where *p*_*i*_ is the *i*-th node of *M*, *f*(*p*_*i*_) is the value of the function *f* at the node *p*_*i*_, *w*_*ij*_ is the weight of the connection between the nodes *p*_*i*_ and *p*_*j*_, *d*_*i*_ is the area weight assigned to the node *p*_*i*_ and *N(i)* is the set of indices corresponding to the direct (also called “1-ring”) neighbors of *p*_*i*_.

The stiffness maxtrix B contains the coefficients of the Laplace operator to be applied to the values *f(p*_*i*_*)*. Example for a simple closed mesh with four points *p*_1_,.,*p*_4_ which are all neighbors of one another:

$$\begin{array}{l} i=1,\;N\left(1\right)=\left(2,3,4\right):\\ \Delta f\left(p_{1}\right)=\frac{1}{d_{1}}\underset{j\in \left(2,3.4\right)}{\sum }w_{1j}\left[f\left(p_{1}\right)-f\left(p_{j}\right)\right]\\ =\frac{w_{12}+w_{13}+w_{14}}{d_{1}}f\left(p_{1}\right)-\frac{w_{12}}{d_{1}}\;f\left(p_{2}\right)\\ -\frac{w_{13}}{d_{1}}f\left(p_{3}\right)-\frac{w_{14}}{d_{1}}f\left(p_{4}\right) \end{array}$$

With analogous equations for *f(p*_2_*), f(p*_3_*)*, and *f(p*_4_*)*, one ends up with

$$\begin{array}{l} \;\left(\begin{array}{c} \begin{array}{c} \Delta f\left(p_{1}\right)\\ \Delta f\left(p_{2}\right) \end{array}\\ \begin{array}{c} \Delta f\left(p_{3}\right)\\ \Delta f\left(p_{4}\right) \end{array} \end{array}\right)=B\left(\begin{array}{c} \begin{array}{c} f\left(p_{1}\right)\\ f\left(p_{2}\right) \end{array}\\ \begin{array}{c} f\left(p_{3}\right)\\ f\left(p_{4}\right) \end{array} \end{array}\right),\text{where }B\\ =\left(\begin{array}{cccc} \frac{w_{12}+w_{13}+w_{14}}{d_{1}} & -\frac{w_{12}}{d_{1}} & -\frac{w_{13}}{d_{1}} & -\frac{w_{14}}{d_{1}}\\ -\frac{w_{21}}{d_{2}} & \frac{w_{21}+w_{23}+w_{24}}{d_{2}} & -\frac{w_{23}}{d_{2}} & -\frac{w_{24}}{d_{2}}\\ -\frac{w_{31}}{d_{3}} & -\frac{w_{32}}{d_{3}} & \frac{w_{31}+w_{32}+w_{34}}{d_{3}} & -\frac{w_{34}}{d_{3}}\\ -\frac{w_{41}}{d_{4}} & -\frac{w_{42}}{d_{4}} & -\frac{w_{43}}{d_{4}} & \frac{w_{41}+w_{42}+w_{43}}{d_{4}} \end{array}\right) \end{array}$$

All Laplace-Beltrami operators in this publication can be introduced on the basis of equation (9).

**Figure 1:**
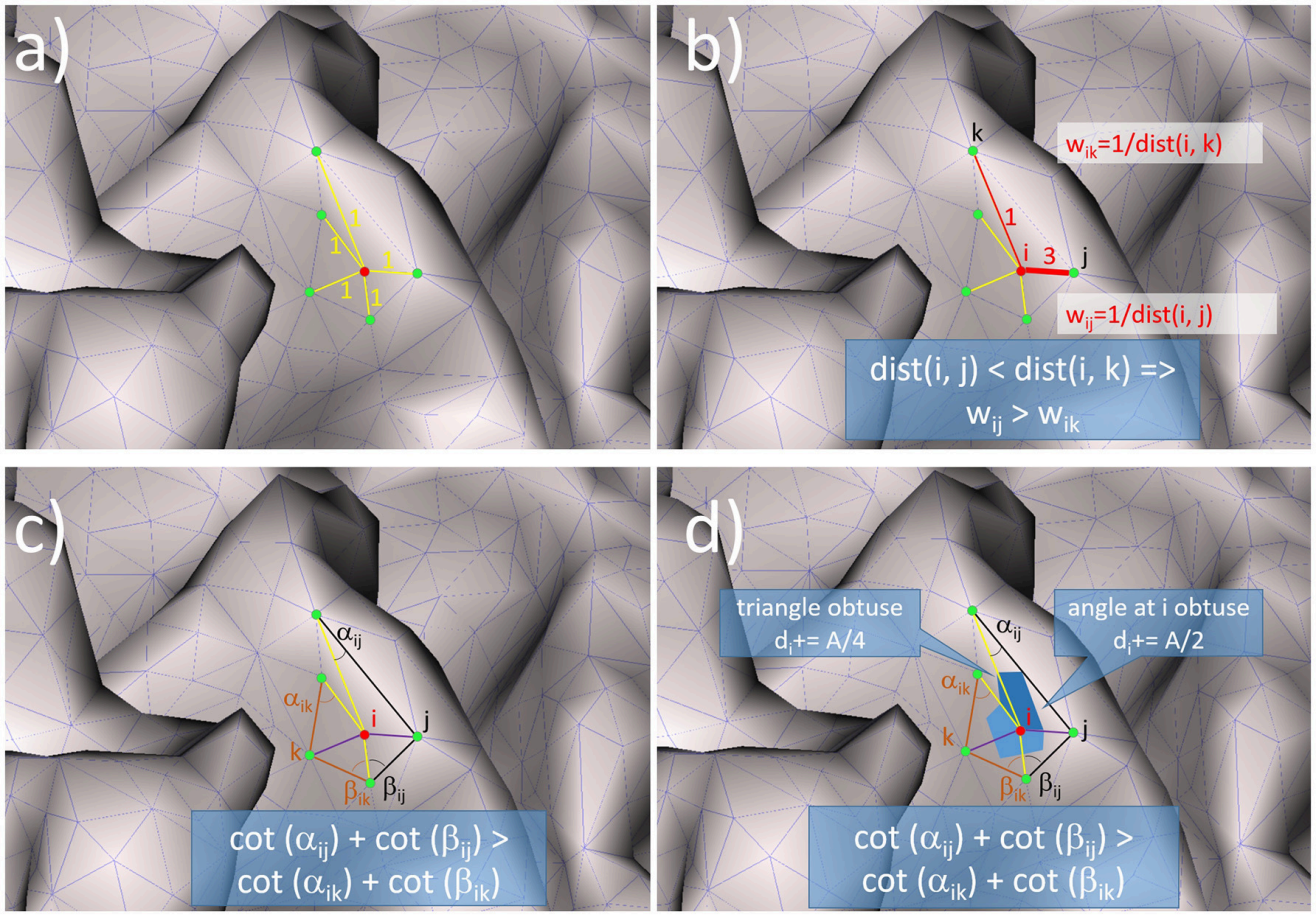
Illustration of the mode of functioning for: **(a)** the unweighted graph Laplacian, **(b)** the weighted graph Laplacian, **(c)** the unweighted geometric Laplacian, **(d)** the geometric Laplacian with mixed area weights.

The unweighted graph Laplacian (UW GrL) (Levy, 2006) is the simplest one. It takes into account only the adjacency of the nodes and not the geometry of the mesh (Figure 1a):

(10) $$\begin{array}{l} w_{ij}=\{\begin{array}{lll} 1 & if & \begin{array}{l} \;p_{i}\;and\;p_{j}\;are\\ directly\;connected \end{array}\\ 0 & otherwise & \; \end{array}\\ \;d_{i}=1 \end{array}$$

The weighted graph Laplacian (W GrL) defined in (Wagner et al., 1996) is similar to the unweighted graph Laplacian but with different weights for the different connections (Figure 1b). In order to represent it in the general form (9) we define the corresponding weights as

(11) $$\begin{array}{r} \begin{array}{c} w_{ij}=\frac{1}{dist\left(p_{i},p_{j}\right)}\\ d_{i}=\underset{j\in N\left(i\right)}{\sum }dist\left(p_{i},p_{j}\right) \end{array} \end{array}$$

where, *dist*(*p*_*i*_, *p*_*j*_) is the distance between the nodes *p*_*i*_ and *p*_*j*_. The geometric Laplacian without area weights (UW GeL) (Pinkall and Polthier, 1993) takes into account not only the connectivity between the nodes but also the geometry of the mesh by including the cotangents of the angles into the weights *w*_*ij*_:

(12) $$\begin{array}{r} \begin{array}{c} w_{ij}=\frac{\cot\left(\alpha_{ij}\right)+\cot\left(\beta_{ij}\right)}{2}\\ d_{i}=1 \end{array} \end{array}$$

where α_*ij*_ and β_*ij*_ denote the two angles opposite to the edge *(i, j)*(Figure 1c). The geometric Laplacian with mixed area weights (W GeL) (Meyer et al., 2003) takes into account not only the angles but also the areas of the triangles in the mesh (Figure 1d):

(13) $$\begin{array}{r} \begin{array}{c} w_{ij}=\frac{\cot\left(\alpha_{ij}\right)+\cot\left(\beta_{ij}\right)}{2}\\ d_{i}=A_{mixed} \end{array} \end{array}$$

Due to (Meyer et al., 2003) the area *A*_*mixed*_ is defined as the Voronoi area if the triangle is not obtuse and in the case of an obtuse triangle the area connected with the midpoint of the edge opposite to the obtuse angle. *A*_*mixed*_ can be calculated with the following algorithm written in pseudocode:

(14) $$\begin{array}{l} A_{mixed}=0\\ \text{For each triangle }T\text{ from the }1-\text{ring neighborhood of }p_{i}\text{ do:}\\ \text{ If }T\;is\;non-obtuse\\ \;A_{mixed}+=\text{Voronoi region of }p_{i}\;in\;T\\ \text{Else}\\ \text{ If the angle of }T\text{ at }p_{i}\text{ is obtuse}\\ \;A_{mixed}+=area(T)/2\\ \text{Else}\\ \;A_{mixed}+=area(T)/4 \end{array}$$

The Voronoi region of ***p***_***i***_ in ***T*** is the set of points in ***T*** such that the distance to ***p***_***i***_ is not greater than the distance to any other two nodes forming the triangle ***T***.

There are many other possible choices for the area around the nodes, however, *A*_*mixed*_ is identified as the best-choice finite-volume region corresponding to the infinitesimal neighborhood on a continuous surface patch (Meyer et al., 2003). The areas of the triangles were calculated with a stabilized Heron's formula (Kahan, 2014) in order to avoid problems for needle-like triangles.

In the following, the operators described by equations (10–13) will be compared with respect to their performance as regularization term for cortical LORETA. Beforehand, it is appropriate to look at some important properties of the Laplace operator (Chung, 1996).

1. The Laplace operator is diagonally dominant
2. The Laplace operator is positive-semidefinite (all eigenvalues are non-negative)
3. There is exactly one eigenvalue which is equal to zero.
4. The Laplacian matrix is singular.

Since the Laplacian matrix is singular it cannot be used directly in equation (7) for calculating the inverse operator *T*. In order to be able to use the Laplace operator into (7) the matrix *B*^*T*^*B* is replaced by its approximation $B^{T}B+\sigma I_{m}$. In that case the weighting matrix *W* obtains a new form

(15) $$\begin{array}{r} W_{cortical}=\left[\Omega \left(B^{T}B+\sigma I_{m}\right)\Omega \right]⊗I_{3}. \end{array}$$

The parameter σ is in the interval $\left(0\;\frac{\eta }{2}\right]$, where η is the largest singular value of the matrix *B*^*T*^*B*.

In order to demonstrate the effect of the parameter σ on the LORETA solution the equation (7) was modified such that depth weighting was not used and only the effect of the Laplacian could be observed. The unweighted minimum norm solution and the unweighted LORETA solution for different σ's were calculated for a simulated bilateral activation of auditory cortex, and compared for the graph Laplacian. A simplified form of the cortex was used for a better visualization of the smoothing effect.

**Figure 2:**
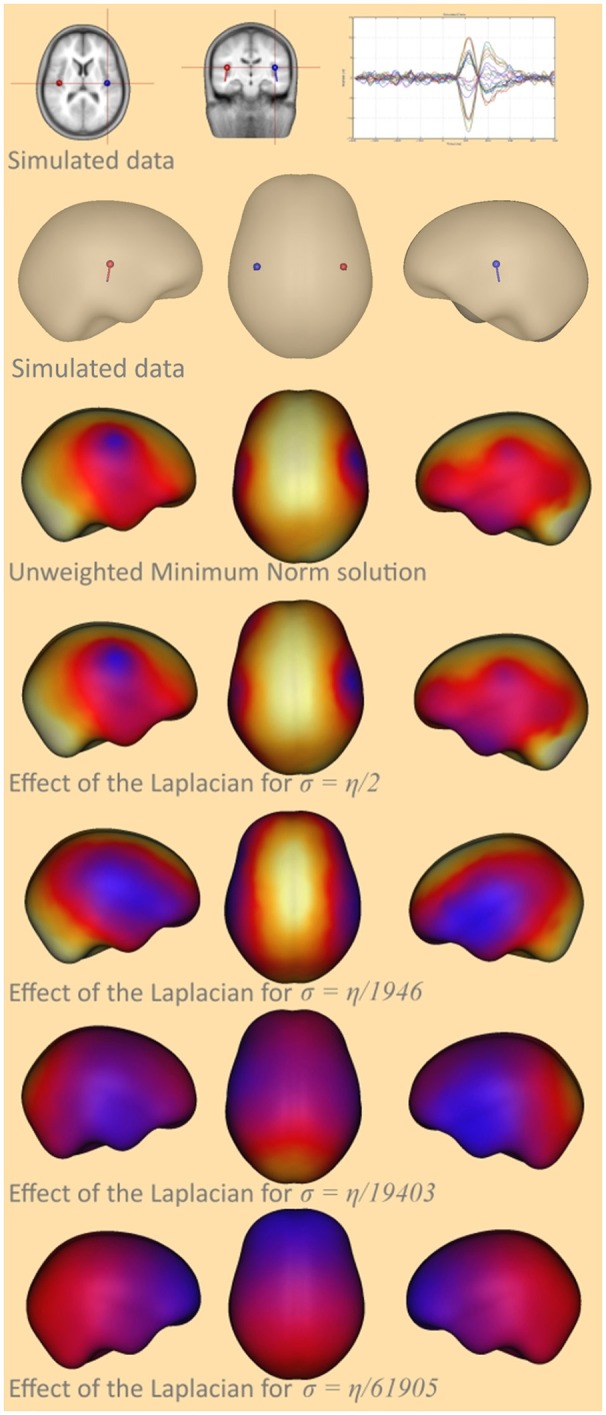
Smoothing effect of the surface Laplacian. The top-most row and the second row show the simulated auditory sources together with a butterfly plot of the signal created at the sensors, the third row shows the unweighted minimum-norm solution, the fourth row the cortical LORETA solution for **σ** = 65, the fifth row for σ = 6.7e-2, the sixth for σ = 6.7e-3, and the bottom-most row shows the cortical LORETA solution for σ = 2.1e-3.

For $\sigma =\frac{\eta }{2}$ the solution with Laplace showed high similarity to the solution without Laplace. This means that the effect of the Laplace operator attenuates for large σ. For σ approaching zero the smoothness of the solution increases, until finally both auditory sources are fused together in the frontal area (Figure 2). This means that the impact of the Laplace operator becomes stronger for smaller values of σ.

Intuitively, this behavior can be explained as follows: when we add large values to the diagonal of the Laplace matrix the diagonal elements become very large compared to the off-diagonal elements and the matrix behaves similar to a diagonal matrix. A diagonal matrix acts merely as a scaling matrix and does not affect the neighboring nodes.

The problem remains how to determine the optimal value for the parameter σ. Until now the knowledge was derived that for too large values of σ the solution with Laplacian “converges” to the solution without Laplacian because of the attenuated effect of the operator and for too small values the smoothing effect is very strong, resulting in a solution which does not match the measured data very well. Consequently, the optimal value should be a trade-off between these two extremes. For that reason two measures are used for estimating the optimal value: the goodness of fit (GOF) and the similarity to the solution without Laplace. The GOF is defined as in Hämäläinen et al. (1993)

(16) $$\begin{array}{r} GOF=100\cdot \left(1-RV\right) \end{array}$$

where, RV is the residual variance and is given by

(17) $$\begin{array}{r} RV=\frac{\sum_{i=1}^{n}{\left(b_{i}-{\hat{b}}_{i}\right)}^{2}}{\sum_{i=1}^{n}b_{i}^{2}}, \end{array}$$

here *b*_1_, ⋯, *b*_*n*_ are the measured data at *n* channels and ${\hat{b}}_{1},\;\cdots \;,{\hat{b}}_{n}$ are the data reconstructed by the linear solver (with or without Laplacian). If *GOF* = 100%, the model explains the data perfectly, whereas *GOF* = 0% means that the model does not match the data at all. The similarity between solutions is calculated using the same equation as for the residual variance with the only difference that *b*_1_, ⋯, *b*_*m*_are the normed source power values (i.e. ϵ[0 1]) for *m* source space points calculated without using a Laplacian and ${\hat{b}}_{1},\;\cdots \;,{\hat{b}}_{m}$are the corresponding values calculated with a Laplacian. A value of 0% would mean that the solutions are identical. Values larger than 100% are also possible.

**Figure 3:**
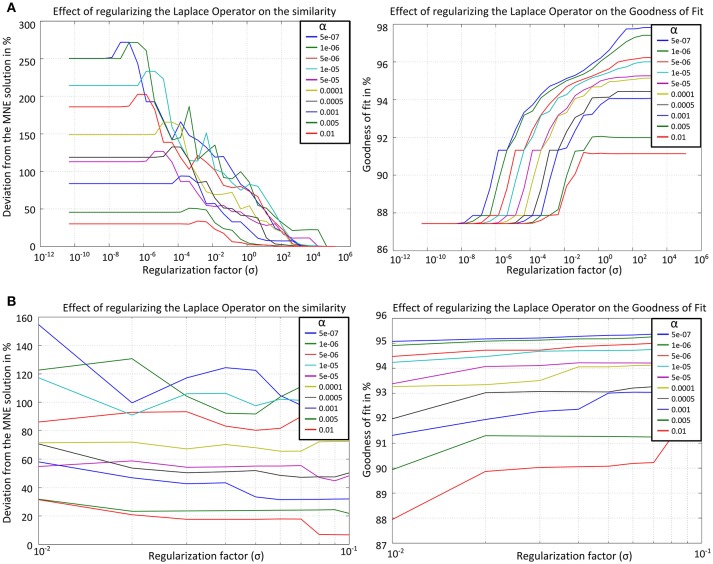
Results for the similarity to the solution without Laplacian (left) and the GOF (right) for the graph Laplacian applied on the simplified cortical form over **(A)** the whole range of **σ** (top row), **(B)** **σ** in the interval [10^−2^ 10^−1^] (bottom row). The curves computed for different α values are denoted by different colors.

In order to derive an optimal value for σ a data set was simulated with 2 bilateral auditory sources and noise from a real EEG measurement such that the signal-to-noise ratio (SNR) was 20. For that data set both measures (GOF and similarity) were calculated for different σ and different α values. The results were plotted as graphs with the x-axis denoting the values for σ and the y-axis denoting either GOF or the similarity to the solution without Laplacian. For each α value one line with different color was added (Figure 3A).

From Figure 3A one can choose an interval for the values of σ in which the trade-off between similarity and GOF is satisfied. At the left it is apparent that for σ > 1 the effect of the Laplacian becomes weaker and the solution is similar (deviation < 5%) to that without Laplacian. In the right figure one can see that in order to get a solution with GOF ≥ 90% the value of σ should not be smaller than 10^−^2. The only value which satisfies the conditions GOF ≥ 90% and deviation to the solution without Laplacian ≥ 10% is σ = 0.04. Since the values in the first calculation were exponentially sampled now a subinterval [10^−2^ 10^−1^] is chosen with linear sampling. Figure 3B shows the results after recalculating both measures for the smaller interval.

**Figure 4:**
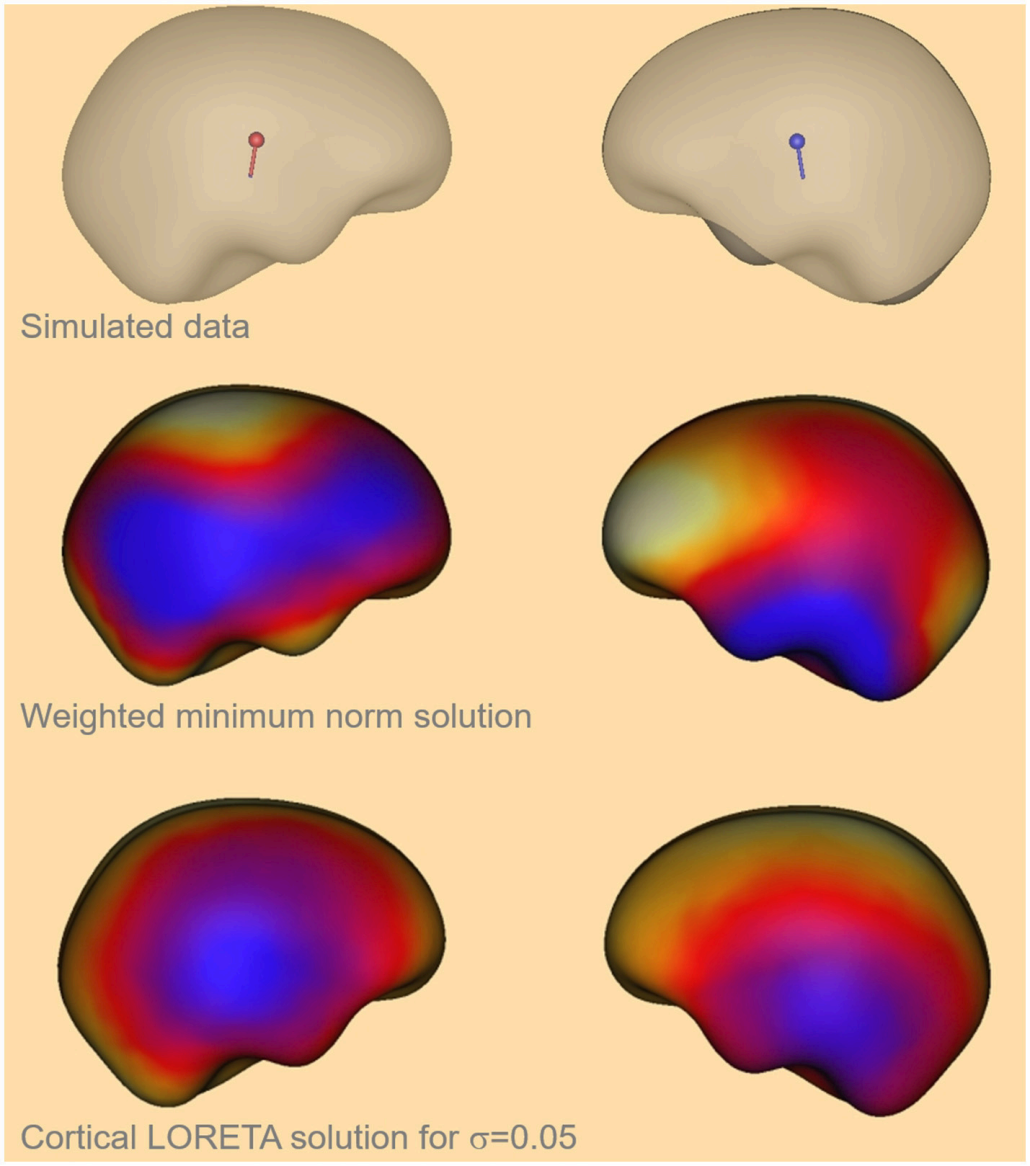
Visual comparison of the simulated auditory sources (top), the depth weighted minimum-norm solution (center) and the cortical LORETA solution (bottom) for the optimal value of σ.

Here, the values [0.07, 0.06, 0.05, 0.04, 0.03] were identified to satisfy the required conditions. The mean value of that interval was taken for further calculations, i.e., σ = 0.05. For that value the GOF is larger than 90% for all α and the deviation from the solution without Laplacian is larger than 17%. The cortical LORETA result for the optimal value is shown in Figure 4 together with the corresponding solution without a Laplacian for a simulated data set with two bilateral auditory sources.

In Figure 4 one can see that the cortical LORETA solution is not more widely distributed than the solution without a Laplacian. Furthermore, the regions with maximal activity are shifted toward the simulated activity regions which are shown at the top of the figure.

This procedure was repeated for all four Laplace operators and for three different types of cortical meshes.

### Anatomical MRI data

**Figure 5:**
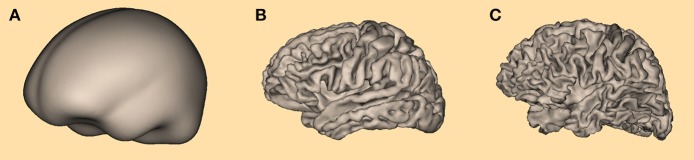
Three different cortical meshes were used for determining the optimal value for **σ**: **(A)** the most simple form with 750 nodes, **(B)** averaged cortex form with 3,709 nodes, and **(C)** individual cortex form with 3,973 nodes.

The procedure for determining the optimal value for σ was applied for three types of cortical meshes (Figure 5): a simplified cortical mesh with 750 nodes, a more realistic cortical form with 3,709 nodes resulting from a non-linear co-registration of 10 individual MRI data sets, and an individual cortical mesh with 3,973 nodes.

The simplified cortical surface was extracted from an averaged MRI created based on 50 T1-weighted MRIs linearly aligned in Talairach space. An initial, coarse triangular cortical surface was manually deformed to fit to the inner bone surface and finally shrunk by a small amount to approximate the cortex.

For the averaged MRI resulting from the non-linearly co-registered data sets and for the individual MRI data set T1-weighted structural magnetic resonance images (MRI) were obtained from 10 participants using a Philips Gyroscan 1.5 T magnetic resonance imaging system (Philips Medical Systems, Gyroscan ACS-T). The resolution of the MRIs was 256 × 256 × 200 voxels, with 200 slices covering the left-to-right direction (Jordanov et al., 2010).

The non-linearly averaged cortex and the individual cortex were automatically segmented using the BESA MRI 2.0 software (BESA GmbH, Gräfelfing, Germany). Based on the provided T1-weighted MRI, first a voxel-based classification was performed (Zhang et al., 2001). Next, an implicit surface representation of the white matter-gray matter interface was reconstructed from the classification result. The implicit surface was finally slightly inflated to approximate the cortical surface in the middle of the gray matter layer (Chan and Vese, 2001; Lanfer, 2014).

### Simulated data

For the comparison between the different Laplace operators with respect to various performance measures 1,000 cortical sources were simulated (here, the realistic cortex with 3,709 nodes was used), however, colocalization with nodes of the cortical mesh used for the calculation of the leadfields was prevented in order to avoid an inverse crime. In that way 1,000 simulations without noise were generated for 1,000 different source locations. In every simulated data set only one source was active. For all simulations a 4 shell ellipsoidal head model as implemented in the software package BESA Research 6.1 (Berg and Scherg, 1994) was used.

Leadfields were computed for a sensor configuration comprising 127 electrodes on a dense grid in an equidistant layout as defined by the geodesic sensor net (Tucker, 1993).

### Measures for the comparison

The measures used for the comparison were localization error, source depth, depth shift, number of local maxima, residual variance and computation time. For the estimation of the localization error and of the depth shift two different approaches were applied. The first approach was to take the mesh node with the maximal amplitude (MA) as the location of the estimated activity and the second approach was to take the center of mass (COM) as the location of the estimated activity. In both cases the image resulting from the source reconstruction was prepared in such a way that all active nodes with activity below 50% of the maximal activity were set to zero. In that way it was ensured that only the nodes with significant contribution to the source reconstruction were used for the comparison (Lin et al., 2006).

The localization error was defined as the distance between the estimated and the simulated source location (Lin et al., 2006; Lucka et al., 2012). Smaller values for the localization error mean better solution. The source depth was defined as the distance between the source (simulated or estimated) and the nearest sensor. A solution was considered good if the depth of the estimated source was approximately the same as the depth of the simulated source. The depth shift was defined as the difference between the depth of the simulated source and the depth of the estimated source (Lin et al., 2006; Lucka et al., 2012). Positive values for the depth shift mean that the simulated source was deeper than the estimated one.

The number of local maxima was investigated to provide an estimate of how many false positives were found in the estimated solution. A local maximum was defined as a mesh node which had larger amplitude than all of its direct neighbor nodes.

For the statistical comparison of the measures one-way ANOVA was applied as implemented in MATLAB and Statistics Toolbox Release 2012b, The MathWorks, Inc., Natick, Massachusetts, United States. For the pairwise comparison of the measures in a *post hoc* statistical step, Tukey's ‘Honest Significant Difference’ method was used as implemented in R (R Core Team, 2015).

### Identifying an appropriate **α** value

After the optimal value for the parameter σ was determined it was necessary to look for an appropriate α value to be used for the calculations. There are many automatic procedures for doing that, e.g., the L-curve. However, these procedures do not always yield the “best” value. Therefore, another procedure was used here.

The first step was to determine the α value for data without noise. Since in equation (7) the pseudo-inverse is used instead of the inverse, α is not a continuous quantity and can only change with the singular values of the given matrix. For that reason an additional parameter is introduced which determines how many singular values should be set to 0 for the inversion. This parameter, called SVD cutoff index (SCI), can have only integer values between 1 and the number of total singular values. If the value is 1 then only the last (smallest) singular value is set to zero, if e.g., the value is 10 then the ten smallest singular values are set to zero. After the SCI is chosen the corresponding SVD cutoff value (SCV) is chosen to be between the singular value with index SCI and the one with index SCI+1. The value for the parameter α can be calculated as the ratio:

(18) $$\begin{array}{r} \alpha =\frac{SCV}{Largest\;Singular\;Value} \end{array}$$

The procedure of determining the optimal α value is to calculate the estimation once with and once without Laplacian for many different SCIs and then to take the index with the best results with respect to localization error and number of local maxima.

## Results

The first step in the analysis of the different Laplace-Beltrami operators was to determine their optimal values for σ. This was done by the procedure described in the section “LORETA with cortical constraint.” The results are shown in Table 1.

**Table 1:** Optimal values for **σ**.

	**UW GrL**	**W GrL**	**UW GeL**	**W GeL**
Simplified cortex (750 nodes)	0.05	1.1E-7	0.0095	1.5E-6
Realistic cortex (3709 nodes)	0.025	1.5E-7	0.0065	3.5E-6
Individual cortex (3973 nodes)	0.055	2.2E-7	0.003	3.0E-6

In the case of individual cortex, for two of the values for α (0.01, 0.005) there were no acceptable σ values for any of the weighted graph Laplacian, unweighted geometric Laplacian and weighted geometric Laplacian. Therefore, the maximal α value used in these cases was 0.0025. The values in Table 1 for σ were used for further calculations.

In the following, the optimal value for α in the case of data without noise and for the case of the realistic cortex with 3,709 nodes was determined. The source estimations were calculated with and without Laplacian for one randomly selected dataset from the simulated data without noise for SCIs 1, 20, 40, 60, 80, and 100. For every SCI the localization error (point with max. amplitude) and number of local maxima were calculated. The results are shown in Table 2.

**Table 2:** Table for determining the optimal value for α in the case of data with no noise and for the realistic cortex with 3,709 nodes.

**SCI:**	**1**	**20**	**40**	**60**	**80**	**100**
SCV: NoL	3.5E-9	9.4E-6	1.2E-4	3.5E-4	8.7E-4	1.8E-3
Alpha: NoL	9.9E-13	2.7E-9	3.4E-8	1.0E-7	2.5E-7	5.2E-7
Localization error (MA): NoL	14.7	15.0	15.4	15.7	15.8	16.1
Number of local maxima: NoL	5.4	5.6	5.7	5.9	6.2	6.3
SCV UW GrL	1.1E-9	3.4E-6	4.1E-5	1.6E-4	4.6E-4	1.3E-3
Alpha UW GrL	7.5E-15	2.4E-11	2.9E-10	1.2E-9	3.3E-9	8.8E-9
Localization error (MA): UW GrL	11.4	11.5	11.7	12.0	12.3	12.7
Number of local maxima: UW GrL	4.5	4.7	4.8	4.8	4.9	4.9

From Table 2 one can see that the localization error and the number of local maxima for both using no Laplacian and for unweighted graph Laplacian, are best for the smallest SCI, in that case 1. Consequently, the α-value for the further calculation was chosen to be 9.9e-13 for the operator without Laplacian and 7.5e-15 for the operator with the unweighted graph Laplacian.

It was assumed that the behavior of the inverse operator with the remaining Laplacians would be similar to those shown in Table 2. Therefore, for all Laplacians only the smallest singular value was set to zero. This resulted in the following α values: α_NoL_ = 9.9E-13, α_UW_ _GrL_ = 7.5E-15, α_W__GrL_ = 5.2E-15, α_UW_ _GeL_ = 6.5E-15, α_W__GeL_ = 6.3E-15. These values were used for further calculations.

The first measure investigated for the accuracy of LORETA with different Laplace operators was the correlation of the estimated source depth with the simulated source depth. This correlation was computed for two different approaches: once the center of mass (COM) was taken as the point for the estimated source, and once the grid point with the maximal amplitude (MA) was taken. The correlation coefficients for the different Laplace operators are depicted in Table 3.

**Table 3:** Correlation coefficients for the connection between the depth of the simulated sources and the depth of the estimated sources.

	**NoL**	**UW GrL**	**W GrL**	**UW GeL**	**W GeL**
COM	0.8658	0.9051	0.895	0.9057	0.8989
MA	0.7295	0.8131	0.8066	0.7945	0.782

**Figure 6:**
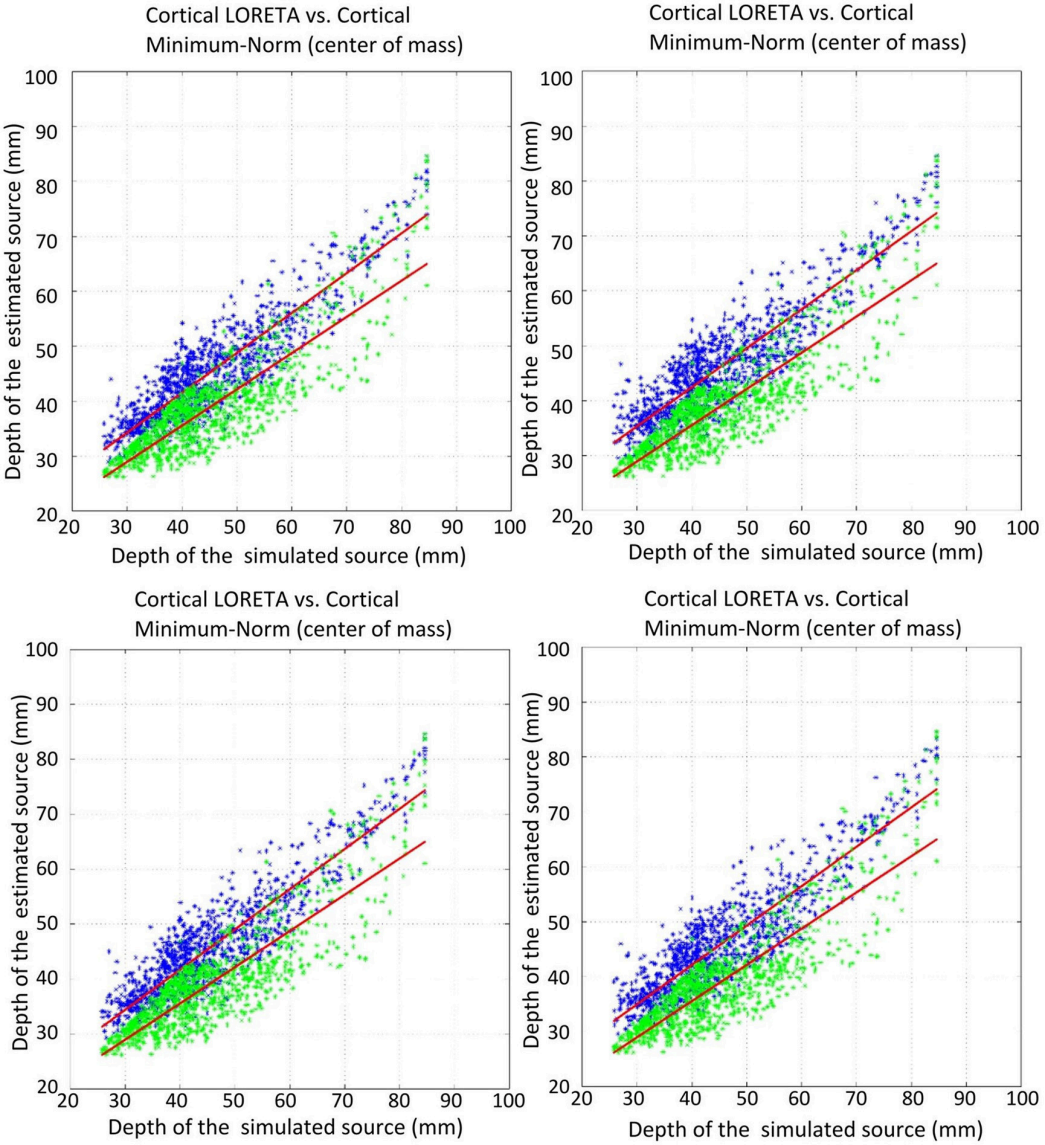
Correlation of the estimated source depth (center of mass) with the simulated source depth. Green shows minimum-norm, blue depicts LORETA. The plots show: top left, unweighted graph Laplacian; top right, weighted graph Laplacian; bottom left, unweighted geometric Laplacian; bottom right, weighted geometric Laplacian.

**Figure 7:**
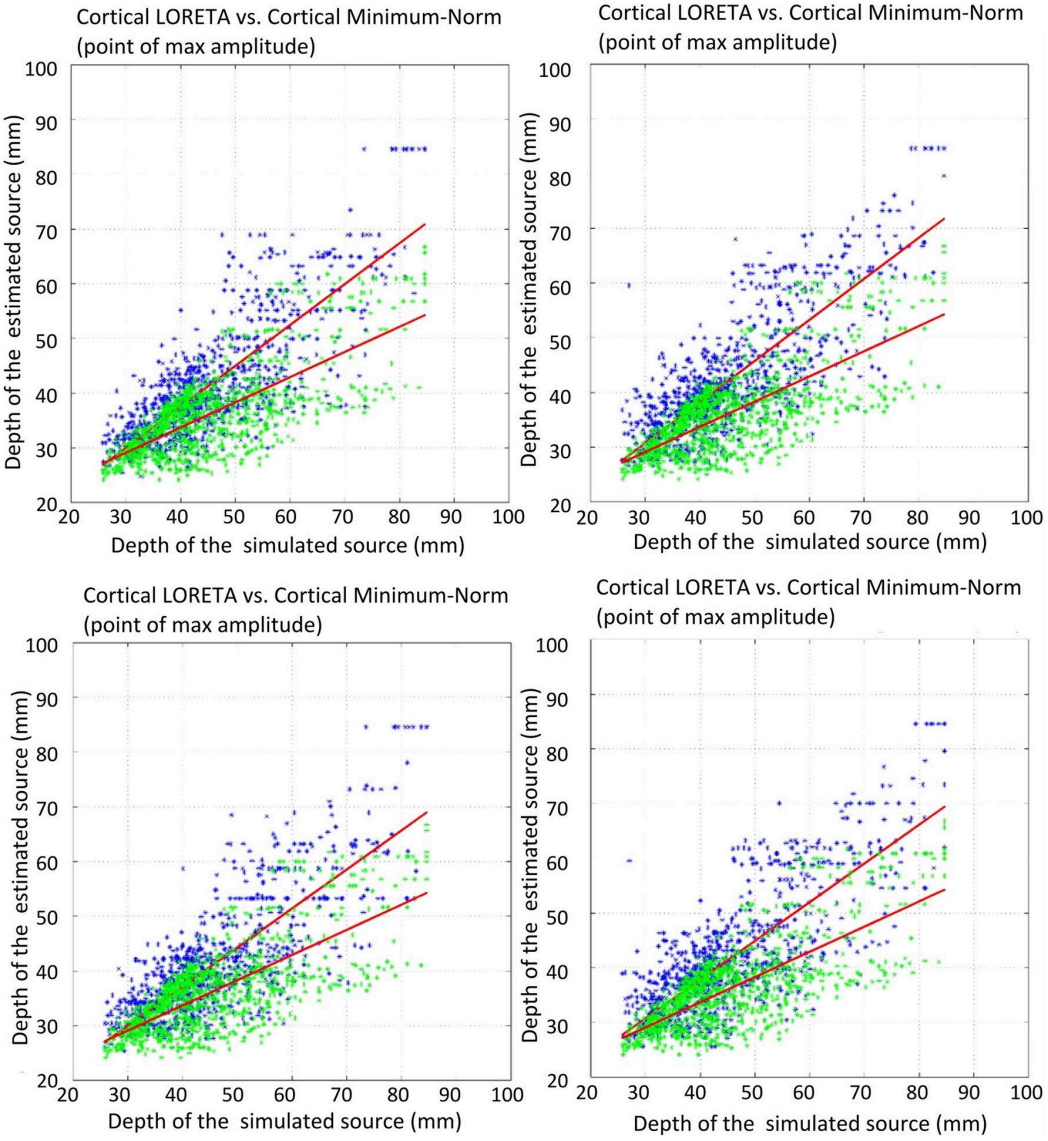
Correlation of the estimated source depth (point of max amplitude) with the simulated source depth. Green shows minimum-norm, blue depicts LORETA. The plots show: top left, unweighted graph Laplacian; top right, weighted graph Laplacian; bottom left, unweighted geometric Laplacian; bottom right, weighted geometric Laplacian.

The comparison of the regression slopes of the source depth by the mean of one-way analysis of covariance (ANCOVA) resulted in statistically significant differences in both cases: COM (F(4, 4990) = 6.637, *p* < 0.0001), and MA (F(4, 4990) = 53.52, *p* < 0.0001). The *p*-values obtained for the pairwise comparison of the slopes are shown in Table 4. Figures 6, 7 visualize the data together with the corresponding regression lines.

**Table 4:** *P*-values for the pairwise comparison of the slopes for the source depth.

**Comparison**	**Slopes comparison (COM)**	**Slopes comparison (MA)**
NoL vs. UW GrL	4.4e-05	0.00
NoL vs. W GrL	0.002	0.00
NoL vs. UW GeL	8.3e-06	0.00
NoL vs. W GeL	0.0004	0.00
UW GrL vs. W GrL	0.33	0.89
UW GrL vs. UW GeL	0.69	0.16
UW GrL vs. W GeL	0.58	0.10
W GrL vs. UW GeL	0.17	0.13
W GrL vs. W GeL	0.67	0.08
UW GeL vs. W GeL	0.34	0.82

There was no statistically significant difference between the slopes of the different Laplace operators. However, it emerged that the slopes for the source depth correlation in the case of reconstruction without Laplacian were statistically different from the slopes in the cases with Laplacian (Table 4).

**Figure 8:**
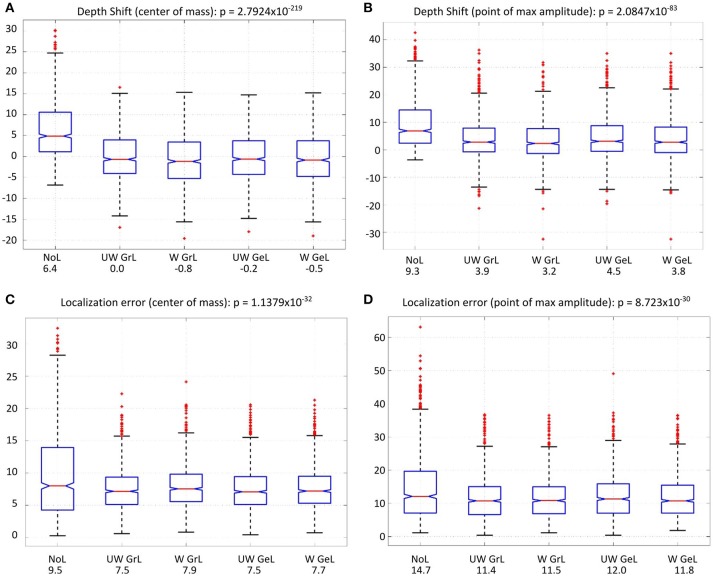
Comparison of the depth shift [top row **(A,B)**] and the localization error [bottom row **(C,D)**] for all four Laplace operators and without a Laplacian (left-most). The red lines on each box mark the median, the blue boxes denote the 25th and 75th percentiles, the dashed lines extend to the most extreme data points not considering outliers; red crosses denote the outliers. The values beside the captions on the x-axis are the mean values in millimeter. **(A)** depth shift (center of mass), **(B)** depth shift (point of max amplitude), **(C)** localization error (center of mass), **(D)** localization error (point of max amplitude).

In order to statistically investigate the different Laplace operators with respect to the depth bias the mean values of the depth shifts were compared (Figures 8A,B).

For the depth shift there was a statistically significant difference between the results using different Laplace operators and no Laplace at all as determined by one-way ANOVA for both COM[F_(4, 4995)_ = 282.52, *p* < 0.0001] (Figure 8A) and MA [F_(4, 4995)_ = 101.74, *p* < 0.0001] (Figure 8B). A Tukey *post-hoc* test revealed that the source estimations without Laplacian showed significantly larger depth shift than the source estimations with all other Laplacians (*p* < 0.0001) (Table 5, columns 4 and 5). Between the Laplace operators only the depth shift with the unweighted graph Laplacian was significantly different from the one with the weighted Laplace operator in the case of COM (*p* = 0.009); all other pairwise comparisons did not yield significant differences (Table 5, column 4). In the case of MA the difference was significant only between the weighted graph Laplacian and the unweighted geometric Laplacian (*p* = 0.002) (Table 5, column 5).

**Table 5:** *Post-hoc p*-values for the comparison of all measures using Tukey's honestly significant difference criterion. All comparisons with *p*-value < 0.05 are considered significant.

**Comparisons**	**Localization error (COM)**	**Localization error (MA)**	**Depth shift (COM)**	**Depth shift (MA)**	**Number of local maxima**	**Residual variance**
NoL vs. UW GrL	<1E-7	<1E-7	<1E-7	<1E-7	<1E-7	<1E-7
NoL vs. W GrL	<1E-7	<1E-7	<1E-7	<1E-7	<1E-7	<1E-7
NoL vs. UW GeL	<1E-7	<1E-7	<1E-7	<1E-7	<1E-7	<1E-7
NoL vs. W GeL	<1E-7	<1E-7	<1E-7	<1E-7	<1E-7	<1E-7
UW GrL vs. W GrL	0.1261160	0.9980268	0.0093079	0.1947429	<1E-7	<1E-7
UW GrL vs. UW GeL	0.9999201	0.3710556	0.9197429	0.5541308	<1E-7	<1E-7
UW GrL vs. W GeL	0.8204365	0.6748003	0.1897860	0.9968678	<1E-7	<1E-7
W GrL vs. UW GeL	0.1707762	0.5654501	0.1043073	0.0022440	0.0000018	<1E-7
W GrL vs. W GeL	0.6982818	0.8474643	0.8045523	0.3657272	0.9464850	<1E-7
UW GeL vs. W GeL	0.8830171	0.9893484	0.6669225	0.3390203	0.0000728	<1E-7

The comparison of the localization error yielded also statistically significant results for both COM [F_(4, 4995)_ = 39.57, *p* < 0.0001] (Figure 8C) and MA [F_(4, 4995)_ = 36.1, *p* < 0.0001] (Figure 8D). The *post hoc* test revealed a difference only between the source reconstructions without Laplacian and the source reconstructions with all Laplacians used (*p* < 0.0001) (Table 5 columns 2 and 3).

**Figure 9:**
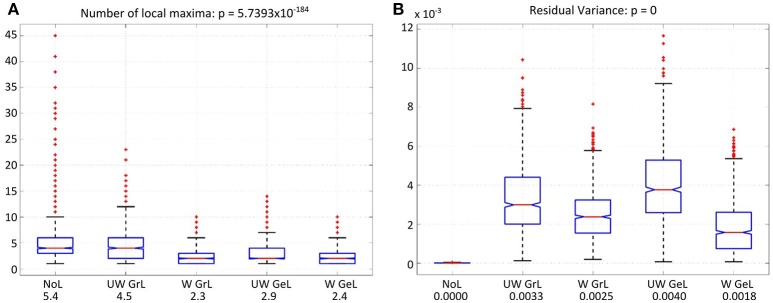
Number of local maxima **(A)** and residual variance **(B)** for LORETA with the different operator types. The red lines on each box mark the median, the blue boxes denote the 25th and 75th percentiles, the dashed lines extend to the most extreme data points not considering outliers; red crosses denote the outliers. **(A)** Number of local maxima for all four Laplace operators and minimum-norm (without Laplacian, left-most). The values beside the captions on the x-axis are the mean number of local maxima. **(B)** Residual variance for all solutions with and without Laplace operator. The solution without Laplace (left-most) yielded the lowest residual value. The values beside the captions on the x-axis are the corresponding mean residual variances.

Another measure was the number of the local maxima as an indicator for false positives in the solution. Again the comparison manifested statistically significant results [F_(4, 4995)_ = 233.37, *p* < 0.0001] (Figure 9A). Additionally, all but one of the pairwise comparisons were significant too (Table 5 column 6). The exception was the comparison of the weighted graph and weighted geometric Laplacians (*p* = 0.95). These two operators manifested a smaller number of local maxima than any other Laplace operators.

For the residual variance also a statistically significant difference was manifested [F_(4, 4995)_ = 1083.76, *p* = 0.0] (Figure 9B). The solution without Laplacian yielded the lowest residual variance compared to the solutions with Laplace operator. The weighted geometric Laplacian revealed the lowest residual variance of the Laplacians.

**Figure 10:**
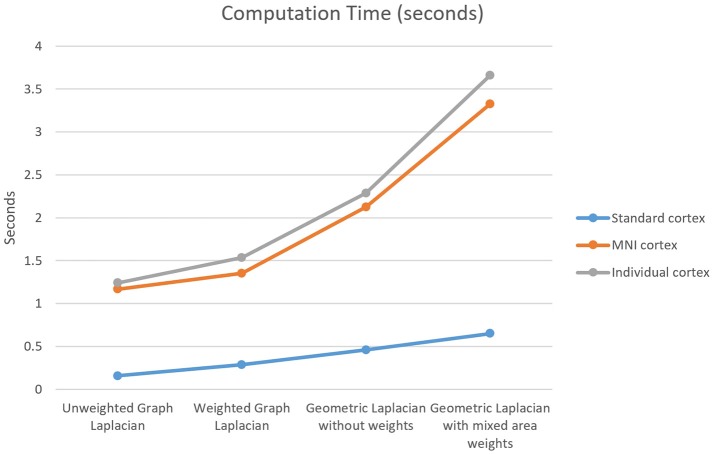
Computation time for the four Laplace operators and three different cortical meshes. The standard cortex contained 750 nodes, the MNI cortex contained 3,709 nodes, and the individual cortex contained 3,973 nodes.

The last measure used for the comparison between the Laplace operators was the computation time in Matlab. The computation time investigated considered only the Laplace operator steps, not the computation time for the inverse operator or for the final solution. The main trend was that the graph Laplacians were calculated faster than the geometric Laplacians (Figure 10).

## Discussion

The aim of this study was to introduce the source localization method LORETA with a cortical constraint and identify an appropriate Laplace-Beltrami operator for optimizing the source reconstruction. The operators in this study were chosen in such a way that they represent an entire family of operators, have sparse structure and operate on a triangular mesh. These operators comprised the unweighted graph Laplacian, the weighted graph Laplacian, the unweighted geometric Laplacian and the weighted geometric Laplacian. The selected operators were investigated with respect to different measures, namely localization error, depth shift, number of local maxima, residual variance and computation time. The optimal solution of the current problem would be the one which minimizes the investigated measures.

For the measures involving location of estimated activity two approaches were followed: using the center of mass (COM) as an estimated source activity location, or using the mesh point with the maximal activity (MA). These two approaches are both common for simulation studies and both have their advantages and drawbacks. The mesh node with the maximal amplitude seems to be more intuitive from the view of the experimental scientist who visually identifies the location of the estimated activity by means of a color map. The idea of using the center of mass follows the line of thought that the solution gained with an l2-norm based linear inverse solver is very smooth and widely distributed, and thus, taking only the maximum of this solution does not take into account the distributed nature of the solution. Since these two approaches yielded different values for the performance measures in this study, both of them were included in order to produce results comparable with other publications using only one of these approaches.

One major property of the minimum norm solution without a Laplacian is that it has a bias toward superficial sources (Skrandies et al., 1995; Lin et al., 2006). It was shown that even the usage of an additional depth weighting term in the linear operator was not always successful in compensating for that bias, and suggested that also the Laplace operator can contribute to the correction of that bias (Skrandies et al., 1995). Therefore, the depth of the estimated sources was investigated in order to be able to detect if there is an advantage in using the Laplace operator with respect to the depth bias. The measures used in this study for the investigation of the depth bias influence were the correlation of the simulated sources depth with the estimated sources depth, as well as the depth shift. It was manifested that using the Laplacian in the inverse operator improved the correlation of depth between simulated and estimated sources, thus confirming previous findings in the literature about the 3D Laplacian (Skrandies et al., 1995; Pascual-Marqui, 1999) to be valid also for the cortical case. However, there was not a significant difference between the various operators. A similar result was observed also for the depth shift where all solutions using the Laplace operator performed significantly better than the solution without Laplace, however, none of the operators was superior to the others.

Contrary to our expectations to find significant differences in the performance of cortical LORETA with respect to the different Laplace-Beltrami operators, no operator emerged to be superior to the others. One possible reason for that behavior could be, that the triangular meshes used for discrete representation of the cortical surfaces were rather regular (or “not irregular enough”), even in the case of individual cortices, and applying operators with weights aimed to correct for irregularity of the meshes did not significantly improve the solution. Another possibility for the missing differences could be the relatively low resolution of the l2-norm solution of the inverse problem. Testing the performance of the source activity estimation using an iterative method like CLARA (Jordanov et al., 2014; Beniczky et al., 2016) could provide a different insight into the source reconstruction capabilities using a Laplace-Beltrami-operator.

A standard measure for the performance of a source estimation method is the localization error. The main question to be answered was how reliable is the result. Again, for this measure, the results did not indicate one Laplacian which performed better than all others, however, using a Laplacian was again shown to be advantageous for the source estimation.

A completely different approach for the comparison of the solutions was to explore the number of false positives in the estimations. It was known that the simulations contained only one active source and it was expected that the estimated solutions contained also only one source. For the investigation of this assumption, the number of local maxima was considered to be an appropriate measure. In all cases the number of estimated source locations was higher than the number of simulated sources. Again the application of a Laplace operator yielded less local maxima than in the case without Laplace. This finding can be explained by the smoothing effect of the Laplacian, which leads to a reduction of high-frequency spatial components in the reconstructed source activity.

With respect to this measure, two of the Laplace operators manifested a significantly smaller number of local maxima than the other Laplacians. These were the weighted graph and the weighted geometric Laplacians.

The operator which best minimized the residual variance was the weighted geometric Laplacian. However, it performed not as well with regard to this measure as the case without Laplace operator. The solution without Laplacian was expected to yield lower residual variance than the solutions with Laplacian, since LORETA minimizes not only the norm of the solution and the difference between the modeled and the measured data but also strives for a smooth cortical solution. This is an additional condition which has to be fulfilled and this is done at the cost of increased residual variance.

The last measure used for the comparison was the computation time. The main finding here was that the graph Laplacians are calculated faster than the geometric Laplacians. The fastest operator was the unweighted graph Laplacian and the slowest one was the weighted geometric Laplacian. The maximal computation time was less than 4 s for the used meshes, consequently, it was not considered as a crucial measure for the choice of the operator. However, if in the future considerably finer grids are used the computation time is going to be an important factor for the decision.

Regarding all measures investigated in this simulation study, one can conclude that it is generally preferable to use a Laplacian in the linear inverse operator. Methods with Laplacian yielded better results for all used measures except for the residual variance. The identification of one best Laplace operator turned out to be nontrivial, since there was no operator which performed considerably superior to all others. However, two of the operators (weighted graph Laplacian and weighted geometric Laplacian) manifested a smaller amount of false positives than the others, which was considered as an advantage and, consequently, these two operators can be identified as the best candidates for LORETA with cortical constraint.

### Limitations of the current study and future direction

Although this study was designed to be complete and self-contained, it was not possible to take into account all possibly relevant aspects. Since there are many different discretizations of the Laplace operator, it was not possible to investigate all of them. For further types of Laplace-Beltrami operators please refer e.g., to Reuter et al. (2009), Dakov and Venkov (2014). The measures used for the comparison of the operators were applied only for simulated data without noise. Additional investigation of the operators' behavior in the presence of different signal-to-noise ratios would be an interesting topic of further studies. An application of cortical LORETA on measured EEG or MEG data and a comparison study with other, more recent localization methods, would be meaningful after the best operator has been determined. However, this is out of the scope of the current study and worth of future investigation.

Additionally, it is important to mention that there exists an alternative formulation of the linear inverse operator T:

$$\begin{array}{l} T_{2}={\left[L^{T}L+\alpha W\right]}^{-1}L^{T} \end{array}$$

which is equivalent to the operator given by equation (6) (Hansen, 1998). The difference between T given by equation (6) and T_2_ is that T_2_ can be calculated also for a singular matrix W. In the case of cortical LORETA the discrete Laplace operator is always singular. Consequently, the factor α would fulfill two tasks simultaneously:

1. The balance between minimizing the residual norm and minimizing the regularization term and
2. Correcting the rank of the matrix to be inverted.

If we choose a value for α, which is more appropriate for task 1), e.g., 0.005, then this value would be far too high for correcting the rank of the Laplace operator. Consequently, the effect of using the Laplacian would be extremely reduced, resulting in a solution more similar to Minimum norm than to LORETA. If, on the other hand, we choose α in such a way that it is more appropriate for correcting the rank of the Laplacian, e.g., 1e-7, then the Laplacian's rank is going to be corrected but the balance between minimizing the residual norm, and minimizing the regularization term, is not going to be optimal. α and σ depend on different data (σ is dependent on the mesh, whereas α is dependent on the functional data - EEG), thus it cannot be guaranteed that a value for α which is appropriate for both purposes always exists. These considerations led to our idea to introduce the additional factor σ. Furthermore, we use σ only for the discrete Laplacian instead of for the entire matrix W, since all other matrices participating in the calculation of W are regular; changing their singular values using σ would distort the solution.

## Author contributions

All authors listed have made a substantial, direct and intellectual contribution to the work, and approved it for publication.

### Conflict of interest statement

HB is an employee of BESA GmbH, a company which develops and provides software tools for EEG and MEG data analysis. TS and MS are employees and shareholders of BESA GmbH. The remaining authors declare that the research was conducted in the absence of any commercial or financial relationships that could be construed as a potential conflict of interest.
